# Intensive, Real-Time Data Collection of Psychological and Physiological Stress During a 96-Hour Field Training Exercise at a Senior Military College: Feasibility and Acceptability Cohort Study

**DOI:** 10.2196/60925

**Published:** 2024-10-18

**Authors:** Rachele Pojednic, Amy Welch, Margaret Thornton, Meghan Garvey, Tara Grogan, Walter Roberts, Garrett Ash

**Affiliations:** 1 Department of Health and Human Performance Norwich University Northfield, VT United States; 2 Stanford Lifestyle Medicine Stanford Prevention Research Center Stanford University School of Medicine Palo Alto, CA United States; 3 Friedman School of Nutrition Science and Policy Tufts University Boston, MA United States; 4 Health and Civil Sector Leidos Reston, VA United States; 5 Department of Psychiatry Yale University School of Medicine New Haven, CT United States; 6 Section of General Internal Medicine Yale University School of Medicine New Haven, CT United States; 7 Center for Pain Research Informatics Medical Comorbidities and Education Center (PRIME) VA Connecticut Healthcare System West Haven, CT United States

**Keywords:** biomarker, biometric, heart rate variability, saliva, feasibility, warfighter, field training, acceptability, wearable biosensors, real-time, data collection, physiological stress, training exercise, pilot study, sweat sensors

## Abstract

**Background:**

Poor physical fitness, stress, and fatigue are factors impacting military readiness, national security, and economic burden for the United States Department of Defense. Improved accuracy of wearable biosensors and remote field biologic sample collection strategies could make critical contributions to understanding how physical readiness and occupational stressors result in on-the-job and environment-related injury, sleep impairments, diagnosis of mental health disorders, and reductions in performance in war-fighters.

**Objective:**

This study aimed to evaluate the feasibility and acceptability of intensive biomarker and biometric data collection to understand physiological and psychological stress in Army Reserved Officer Training Corps cadets before, during, and after a 96-hour field training exercise (FTX).

**Methods:**

A prospective pilot study evaluated the feasibility and acceptability of multimodal field data collection using passive drool saliva sampling, sweat sensors, accelerometry, actigraphy, and photoplethysmography. In addition, physical fitness (Army Combat Fitness Test), self-reported injury, and psychological resilience (Brief Resilience Scale) were measured.

**Results:**

A total of 22 cadets were included. Two were lost to follow-up due to injury during FTX, for a retention rate of 91%. Assessments of performance and psychological resilience were completed for all remaining participants, resulting in 100% testing adherence. All participants provided saliva samples before the FTX, with 98% adherence at the second time point and 91% at the third. For sweat, data collection was not possible. Average daily wear time for photoplethysmography devices was good to excellent, meeting a 70% threshold with data collected for ≥80% of person-days at all time points. Of the participants who completed the FTX and 12 completed a post-FTX acceptability survey for a response rate of 60%. Overall, participant acceptance was high (≥80%) for all metrics and devices.

**Conclusions:**

This study demonstrates that wearable biosensors and remote field biologic sample collection strategies during a military FTX have the potential to be used in higher stakes tactical environments in the future for some, but not all, of the strategies. Overall, real-time biometric and biomarker sampling is feasible and acceptable during field-based training and provides insights and strategies for future interventions on military cadet and active-duty readiness, environmental stress, and recovery.

## Introduction

Poor physical fitness, stress, and fatigue are factors impacting military readiness, national security, and economic burden for the United States Department of Defense [1,2]. Specifically, low physical activity and physical fitness are highly correlated with musculoskeletal injuries (MSKIs). According to a recent report, MSKIs are now considered the greatest medical impediment to military readiness, with the total direct medical cost of treating MSKIs among military trainees alone estimated to be approximately US $15 million per year [2]. Occupational stressors also result in on-the-job and related injuries [2,3], sleep impairments, diagnosis of mental health disorders, and reductions in performance [4]. In response to this human and economic burden, the Department of Defense concluded that the military health system must “reshape its focus on disease and injury treatment and prevention…for optimal human performance in a technology-rich battle space” [5].

Wearable biosensors and remotely collected biologic samples have gained traction and accuracy and could make contributions to this effort. However, field data collection in the trainee and active-duty war-fighter populations requires careful consideration as techniques and devices must be flexible, wearable, durable, and capable of surviving both field training and combat.

Controlled studies have been implemented successfully in the laboratory to examine war-fighters and others (ie, police, firefighters, and first responders) with physical fitness requirements coupled with potential exposure to life-threatening situations [6], including temperature regulation [7], hydration [8], responses to dietary intervention [9-11], and cognitive responses to stress [12-14]. Data from the field, however, are sparse and inconsistent between populations, devices, methodologies, and outcomes. This is problematic as replication is critical to define best practices and to generate standard operating procedures. Moreover, no current data exist to ascertain whether the use of collection devices or strategies is acceptable to war-fighters in the field. There is a need for clear methodology and evidence to support the feasibility and acceptability of biometric and biomarker collection in the field, which has implications for overall cost and efficacy, as well as war-fighter safety and survival.

The purpose of this study was to evaluate the feasibility and acceptability of intensive biomarker and biometric field data collection. To maximize the future feasibility and accuracy of measuring markers of physiological and psychological stress in active duty and deployed war-fighters, Army Reserved Officer Training Corps (AROTC) cadets were studied before, during, and after a 96-hour outdoor field training exercise (FTX).

## Methods

### Design

A prospective observational study was used to evaluate the feasibility and acceptability of intensive data collection, including passive drool saliva sampling, sweat sensors, accelerometry, actigraphy, and photoplethysmography in AROTC cadets. Physical fitness, injury, and psychological resilience were also measured.

### Participants

Members of the Norwich University AROTC unit in Northfield, VT were recruited in person and through email. Participants were included if they were between 18 and 24 years old, a member of the junior (third year) class, and would be participating in the 96-hour FTX. They were excluded if they had a diagnosed heart condition.

### Field Training Exercise

The FTX is conducted biannually by the AROTC between Paine Mountain, Norwich University, and Camp Ethan Allen Training Site in Vermont. The FTX tests cadets’ physical, tactical, and leadership abilities over a 96-hour period, typically completed in the field. The terrain at Paine Mountain and Camp Ethan Allen Training Site is characterized by steep inclines, dense vegetation, and rugged ground. During the FTX (October 2022), the temperature averaged 49 °F over 3 days, with no precipitation.

### Demographics and Brief Resilience Scale

Upon enrollment, participants completed an internet-based survey (Qualtrics XM), which included sociodemographic data and the Brief Resilience Scale (BRS) [15]. The score range on the BRS is from 1 (low resilience) to 5 (high resilience).

### Device Allocation and Data Collection

**Figure 1:**
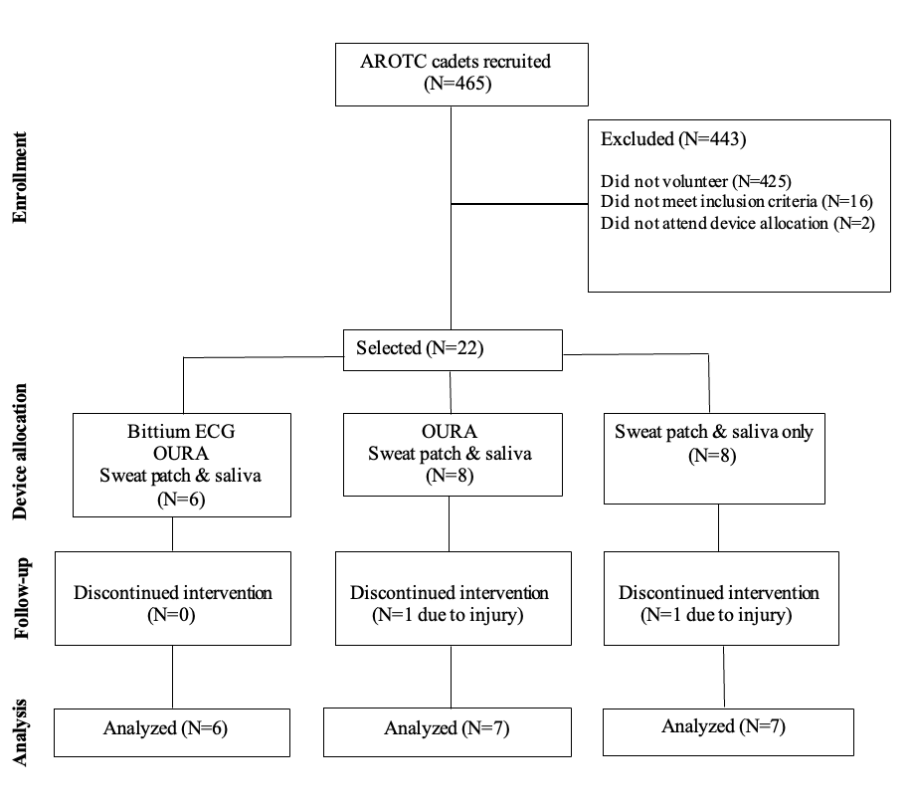
Participant selection, device allocation, and feasibility analysis for a 96-hour field training exercise in Army Reserved Officer Training Corps cadets. AROTC: Army Reserve Officers Training Corps; ECG: electrocardiogram.

One week before the FTX, participant’s height and weight were measured using a calibrated stadiometer and scale and they were provided a passive drool saliva collection kit (Salimetrics). They were then randomized through a simple random number to receive either (1) an ŌURA ring (Generation 2, ŌURA) alone, (2) an ŌURA ring and a clinical-grade portable 3-lead electrocardiogram (ECG; Bittium Faros 180, Bittium Corporation), or (3) no ŌURA or ECG. One day before the FTX, all participants were also assigned a sweat sensor (Nix Biosensors) and asked to charge their ŌURA ring during the day. Device allocation is presented in Figure 1. Data collection time points are presented in Table S1 in Multimedia Appendix 1.

### Saliva Collection

Approximately 2 mL of saliva samples were collected at 3 time points through the SalivaBio Passive Drool Method (Salimetrics): 4 days before FTX, day 1 of FTX, and 10 days after FTX (4 per day for 3 days). A total of 4 samples were collected on each testing day, that are waking, 30 minutes post waking, 11 AM, and 6 PM. Each sample took approximately 5 minutes to collect. Participants provided their samples in a sterile tube, capped their tubes, and placed them in the sample box provided by research assistants. During the FTX, research assistants followed participants in the field to collect and cool samples. Samples were labeled with participant unique identifiers and stored at –20 °C until they were returned to Salimetrics for analysis. All samples were analyzed for cortisol. Day 1 of FTX was also analyzed for cytokines (interleukin [IL]-1, IL-6, IL-8, and TNF-α) at 4 time points.

### Photoplethysmography (ŌURA Ring)

A total of 14 out of 465 participants were selected to wear ŌURA rings fitted according to company specifications to the digit of choice on the nondominant hand. Participants were asked to wear the ring continuously for 7 days before the FTX, during the FTX, and for 10 days after the FTX. They were provided with a charging device and were asked to remove the ring only to charge at specific time periods during the day (not overnight). To sync continuously, participants were asked to download the ŌURA web application and had access to their data during the study. ŌURA outputs were calculated using body metrics (sex, age, body mass, and height). Researchers monitored and downloaded all data through a remote dashboard that was updated at noon daily. Wear time, nocturnal heart rate, respiration (30-second resolutions), and heart rate variability (HRV) were detected indirectly with an infrared sensor that detects a participant’s arterial pulse on the finger using photoplethysmography at 250 Hz. HRV, using interbeat interval data during sleep, was analyzed using the root mean square of the SD of R-R intervals.

### Electrocardiogram

Among the 14 participants selected to wear the photoplethysmography device, 6 also wore a portable ECG device (Bittium Faros 180, Bittium Corporation). The skin of participants was prepped by mild abrasion with an alcohol skin prep pad. The removable adhesive ECG device was applied to the sternum region of the chest. Hypoallergenic adhesive tape was applied over the device, wires, and electrodes. The device was worn for 3 days before the FTX, 96 hours during the FTX, and 24 hours after the FTX. Participants were requested to charge the device before the FTX. Upon completion of data collection, participants returned the device to the laboratory, where data were extracted and recorded. HRV during sleep was the primary ECG measurement and was analyzed using the root-mean-square of the SD of R-R intervals.

### Sweat Sensor

All participants wore a sweat sensor (NIX Biosensors) placed on their skin, located on the mid-bicep area of their nondominant arm. The skin of the participant was prepped by mild abrasion with an alcohol skin prep pad and allowed to dry. The adhesive kinesiology tape sensor was then applied and covered with adhesive tape. The sensor was worn for 96 hours during the FTX. Upon completion of data collection, sensors were returned to the manufacturer, where data were to be extracted. Primary outcomes for sweat sensors included sweat rate (mL/min) and aggregate fluid electrolyte (ie, sodium and potassium) loss through sweat (mg).

### Physical Fitness

The AROTC provided all scores of the most recent Army Combat Fitness Test [16], which had been conducted 3 days before the FTX. Scores included 2-mile run time, plank time, sprint-drag-carry, 3-repetition maximum deadlift, standing power throw distance, and hand-release pushup count.

### Army Reserved Officers’ Training Corps Blue Card (CDT CMD FORM 156-4A-R)

All AROTC units use a “Blue Card” to assess cadets in the field [17]. The blue card is a standardized subjective assessment of leadership in the field and grades attributes of leadership (ie, character, presence, and intellectual capacity), core leader competencies (ie, leads, develops, and achieves), and records of observations and counseling. A blue card was completed by Norwich University AROTC senior (fourth year) members for evaluation of all third-year cadets in leadership positions during FTX training. The blue cards and corresponding scores were collected, deidentified, and then scored.

### Injury

Injury documentation during FTX occurs only if self-reported to AROTC leadership. Reported injury frequency and type were collected and deidentified.

### Feasibility

Feasibility was defined by a retention rate (>80%) and adherence rates (>70%) for the assessment measures [18,19], such as survey responses, saliva collections, sweat collection, and HRV sensor wear. Retention was defined as the percentage of participants who completed the baseline survey and completed the 96-hour FTX. The adherence to saliva sampling was defined as the percentage of the collected saliva sample out of a total of 12 collections (4 per day for 3 days). The adherence to sweat collection was defined as 1 day of valid wear time data collection during the FTX. The adherence to wearing a photoplethysmography device (ŌURA ring) was defined as 70% wear time by individual participants and ≥80% of participants having simultaneous data tracked [18,19].

### Acceptability

After the FTX, participants were invited by email to complete an internet-based acceptability survey (Qualtrics) using questions specific to each device and subjective perceptions of the use of wearables in the field. Responses were answered using a Likert scale (1=strongly disagree and 5=strongly agree). Participants were also asked to provide any feedback through open-ended questions.

### Statistical Analysis

Data were compiled and uploaded to SPSS (version 28.0; IBM Corp) and were reviewed for errors, and evaluated for missingness, outliers, and skewness. Descriptive statistics were used for sample characteristics and data completion rates, with variance reported by SD or IQR as appropriate.

### Ethical Considerations

The study was approved by the Norwich University institutional review board (HHS IORG #0004914, IRB #00005859, and FWA#00013380). Informed consent was obtained from all participants.

## Results

### Participants

A total of 22 cadets were included. Participant characteristics and descriptive statistics are reported in Table 1.

**Table 1:** Demographic data of Army Reserved Officers’ Training Corps participants in a 96-hour field training exercise at a senior military college (n=22).

Characteristics	Outcome
Age (years), mean (SD)	20.4 (0.43)
Sex (male), n	12
Race or Ethnicity (White or Caucasian), n	19
Height (cm), mean (SD)	170.5 (7.1)
Weight (kg), mean (SD)	71.6 (11.2)
BMI (kg/m^2^), mean (SD)	24.84 **(**3.27)
ACFT^a^ score (points out of 600), mean (SD)	495.8 (54.32)

^a^ACFT: Army Reserved Officer Training Corps.

### Feasibility

**Figure 2:**
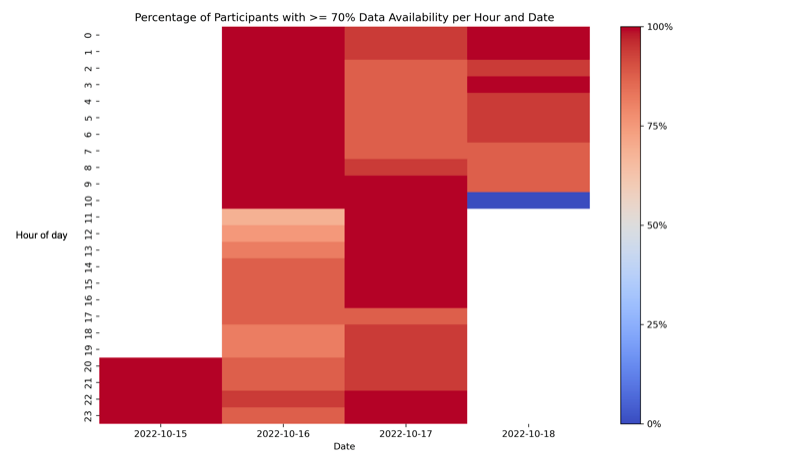
The percentage of Army Reserved Officer Training Corps participants whose plethysmography device (ŌURA ring) detected ≥70% wear time each hour during a 96-hour field training exercise.

Of the 22 cadets that began the FTX weekend, 20 completed the FTX exercises, for a retention rate of 91%. A total of 2 cadets discontinued participation due to injury. Biomarker, performance, ŌURA wear time, and injury data are presented for all participants in Table 2. All participants (N=22) completed 4 saliva samples before the FTX, with 1 sample missed during the FTX due to injury, resulting in a 98% adherence at the second data collection point. A total of 10 days post-FTX, all but 2 participants returned saliva (n=20/22), resulting in 91% adherence at the third data collection point. Performance, as measured by blue card submission, was completed for all participants, resulting in 100% adherence. For all participants that wore ECG and ŌURA rings, electronic datasets were retrieved from the devices for the time period worn. Average daily wear time was good to excellent and left-skewed, meeting the ≥70% threshold [18,19] by all participants during FTX and all but 2 participants during baseline (Table 2). These 2 participants and 8 others had less wear time recorded by the third day of baseline, likely because of lower daytime wear due to participants being instructed to charge their ring on the day before the FTX. The typical charging time for the ring is between 20 and 80 minutes, depending on the starting charge [20]. Figure 2 presents a heatmap illustrating the percentage of participants whose rings detected ≥ 70% wear time each hour. Wear time data for each participant are presented in Figure S1 in Multimedia Appendix 2. No usable data were retrieved from the sweat monitoring devices.

**Table 2:** Completed biomarker and performance data collection variables and average daily ŌURA Ring wear time at baseline and during a 96-hour field training exercise in Army Reserved Officer Training Corps cadets.

Variables			4 Days before FTX^a^ (baseline)		Baseline excluding charging day		FTX
Saliva samples, n (%)			88 (100)		—^b^		87 (99)
Sweat samples, n (%)			—		—		0
Blue card 1, n (%)			—		—		22 (100)
Blue card 2, n (%)			—		—		22 (100)
**ŌURA Ring wearing**							
	ŌURA Ring wear time, median (IQR)	90% (76%-95%)		95% (80%-100%)		96% (93%-97%)	
	Number of participants >90% wear	7		9		12	
	Number of participants 80%-89% wear	2		2		1	
	Number of participants 70%-79% wear	3		3		1	
	Number of participants 65%-69% wear	2		0		0	
	Number of person-days ≥70% wear, n/N (%)	34/42 (81)		25/28 (89)		54/56 (96)	
Injury, n/N			—^b^		—		4/22 (1 shoulder injury, 1 sprained ankle, 1 blunt trauma injury to foot, 1 blunt trauma injury to hand)

^a^FTX: field training exercise.

^b^Not applicable.

### Acceptability

Of the 20 participants who completed the FTX, 12 completed the acceptability survey for a response rate of 60%. Of those that responded, all 12 gave saliva samples; 4 participants used all devices; 3 used 2 devices; and 5 used 1 device. Regarding saliva samples, 90% (11/12) of participants overall reported liking giving samples (>3 on the Likert scale), 89% (11/12) would be willing to give more samples than asked, and 89% (11/12) would be willing to give samples again. However, 75% (9/12) responded that remembering to give samples was difficult. Furthermore, 83% (10/12) of participants disagreed that giving saliva was physically uncomfortable, 50% (6/12) disagreed that it was embarrassing, and 80% (10/12) disagreed that it interfered with sleep. However, 67% (8/12) agreed that providing saliva samples interfered with their regular activities during FTX. Overall, 100% (12/12) of participants enjoyed wearing the ŌURA (>3 on the Likert scale) and 100% would wear it again (>3 on the Likert scale). Regarding the ECG, 100% (12/12) of participants enjoyed wearing the ECG and 100% would wear it again, although 100% agreed that the device interfered with sleep. Regarding the sweat sensor, 86% (10/12) of participants enjoyed wearing the sensor and 86% (10/12) would wear it again. Some adverse experiences were reported with the sweat sensors, with 3 participants reporting skin irritation, 2 participants reporting itching, and 1 participant reporting sweating. Similarly to the sweat sensor, 3 participants who wore the ECG reported skin irritation, 2 reported itching, and 2 reported sweating. There were no adverse experiences reported by participants wearing the ŌURA ring. All acceptability data are presented in Table S1 in Multimedia Appendix 3.

## Discussion

### Principal Results and Comparison With Previous Work

Remote field biologic sample collection strategies and wearable biosensors could contribute to informing training and response models in war-fighters. However, testing in high-stakes active-duty military training settings and deployments is challenging. To maximize the future accuracy of measuring markers of physiological and psychological stress in active duty and deployed war-fighters, we assessed the feasibility and acceptability of using devices and strategies to collect biometrics and biomarkers associated with physiological and psychological stress during a 96-hour FTX in AROTC cadets. We had high retention and adherence (≥90%) rates for all collection protocols. A total of 2 participants were unable to complete the FTX, but this was not due to study observation or device use. Overall, we demonstrated that most, although not all, protocols were suitable for multiday field collection. Specifically, field saliva collection and HRV monitoring through a ring device were feasible; however, sweat collection was not. All methodologies were acceptable to participants, with no safety concerns and few adverse experiences.

While saliva collection has been validated [21] in tactical populations and data collection has been completed successfully during military training in previous studies [11,22,23], this is the first study, to the authors’ knowledge, to examine repeated field testing at multiple time points during the training itself (ie, rather than immediately pre–post or in a structured laboratory setting). It is also the first to assess feasibility and participant acceptability in AROTC cadets while providing saliva samples in the field. Our results mimic those recently reported by Sorenson and colleagues [24], who found a high level of compliance to the free-living salivary sampling protocol, with 95% and 84% of healthy adults being compliant over 3 days. We were successful in 90% participant-administered saliva sample collections 4 times per day, over 3 days as instructed. Participants in this study also reported saliva sampling was not challenging, and they would be willing to provide samples in the field again. However, a majority (67%, 8/12) did note that they felt saliva collection interfered with their activities during the FTX.

With regard to biometric data collection, photoplethysmography devices have been used successfully in war-fighter populations, particularly to determine the risk for COVID-19 infection [25], posttraumatic stress disorder [26], and to monitor sleep and psychological well-being [27]. However, none of these studies examined responses to field stimuli or training, nor did they report outcomes related to feasibility of use in the field or acceptability by users. One previous study examined AROTC cadets during a 31-day advanced camp to understand the future feasibility of using sleep actigraphy in active-duty military populations, although they used a wrist-based monitor [28]. Our results demonstrate that the use of a hand-worn photoplethysmography was feasible in the field, with average daily wear-time good to excellent with a ≥70% threshold [18,19] met by the majority of participants. Furthermore, participants were overwhelmingly supportive (12/12, 100%) of using the device in the FTX setting. Perhaps, most importantly, the wearing of a ring device did not pose significant distraction or safety concerns, with the majority of participants reporting not noticing the device in the field.

With regard to hydration, there is significant interest in the war-fighter population to monitor dehydration and heat stroke [3,29]. Technologies are emerging to enable “detect to treat” opportunities [30], although efficacy and field testing of wearable biomarker monitoring devices remain limited in military populations [31]. In our study, we were attempting to understand whether sweat monitoring can be done over a multiday period and, if so, what the average sweat rate and electrolyte loss were. However, we demonstrated how challenging this endeavor can be and were unable to return any usable data after wearing a sweat sensor for 96 hours in the field. For success in future studies, battery life as well as sweat requirements and storage capabilities for the device should be considered.

### Limitations

There were feasibility challenges and limitations to this study. First, due to the nature of data collection during a highly structured military FTX, a substantial amount of time and effort was placed into planning and coordinating stakeholder groups. This was anticipated, yet still difficult to execute before and during the FTX. Second, due to the extreme hours of data collection (ie, 3 AM) and the rural, mountainous terrain, the protocols were a significant burden on the research team. Furthermore, with regard to saliva collection, due to the field nature of sample collection, exact time stamps were difficult to record and there was a significant burden on research staff due to collection during extreme times, terrain, and weather parameters, which resulted in challenging collection and transport. Third, participants were instructed to collect their first saliva sample immediately upon waking and 30 minutes after waking. Samples were not timestamped, and this could affect the analysis and interpretation of some circadian biomarkers such as cortisol. Fourth, participants were allowed to see their ŌURA data on their phone app, and this could have affected their behavior. To improve future iterations of this type of field collection, all saliva samples would need to be time stamped, either by the participant or electronically. Finally, regarding sweat monitoring, due to low sweat rates combined with long work-to-rest ratios, no usable data were recorded. The devices were designed for sustained endurance activities with continuous sweat collection. There was also a hardware malfunction due to battery life being unable to withstand the 96-hour FTX. As such, these particular devices were determined to not be feasible for this type of activity.

The real-time data biometric and biomarker samples we were able to collect in a field environment may provide insights and strategies for future research and interventions on injury prevention, military readiness, environmental stress, and recovery. In addition, the large datasets returned by the monitoring devices provide the potential for unique analyses and predictive capabilities. While the goal of the study was to use traditional a priori statistical modeling, there is an opportunity to use machine learning with inputs from 24-hour curves of biometrics and biomarkers to derive more comprehensive and objective prognostic models and frameworks for war-fighters in the field. Indeed, this type of analysis is currently being used to understand hydration [32] and sleep [27] in military populations.

### Conclusions

In conclusion, our data highlight unique strategies to combine real-time biomarker and biometric data collection during AROTC field training to offer improved ROTC outcomes while also demonstrating possibilities for predicting or preventing occupational hazards faced by military personnel. The feasibility and acceptability of using remote field biologic sample collection strategies and wearable biosensors during an AROTC cadet field training exercise demonstrate the potential for future use for some, but not all, strategies in higher stakes tactical environments. In the future, similar methods could be used across a wide range of active-duty service members in simulated combat and deployment operations to understand more realistic predictors and consequences of physiological and psychological stress.

## Appendix

Multimedia Appendix 1 Data collection timelines for each variable of interest.

Multimedia Appendix 2 A heatmap illustrating plethysmography device (ŌURA ring) detected wear time ≥ 70% each hour for each participant.

Multimedia Appendix 3 Acceptability of wearable device use in the field. A total of 12 participants completed the acceptability survey and answered using a 5-point Likert scale (1= strongly disagree and 5=strongly agree).

## Abbreviations

AROTC: Army Reserve Officers Training Corps
BRS: Brief Resilience Scale
ECG: electrocardiogram
FTX: field training exercise
HRV: heart rate variability
IL: interleukin
MSKI: musculoskeletal injury

## Data Availability

Supporting reported data and results can be requested from the corresponding author RP.
