# Supplementary material for: Intensive, Real-Time Data Collection of Psychological and Physiological Stress During a 96-Hour Field Training Exercise at a Senior Military College: Feasibility and Acceptability Cohort Study
Source: JMIR Form Res. 2024 Oct 18;8:e60925. doi: 10.2196/60925 (PMC11530722; doi:10.2196/60925)
Supplement: Multimedia Appendix 1 [file formative_v8i1e60925_app1.docx]

**Table S1.**

|  | 4 days prior to FTX^a^ (baseline) | | | | | Day 1 FTX | | | | 10 days after FTX | | |
| --- | --- | --- | --- | --- | --- | --- | --- | --- | --- | --- | --- | --- |
|  | HRV^b^ | Sleep | | Saliva | | HRV | Sleep | Saliva | Sweat | HRV | Sleep | Saliva |
| Overnight (prior) | x | x | |  | | x | x |  |  | x | x |  |
| Waking | x | |  | | x | x |  | x |  | x |  | x |
| Waking +30 mins | x | |  | | x | x |  | x | x | x |  | x |
| 11 AM |  | |  | | x |  |  | x | x |  |  | x |
| 4 PM |  | |  | | x |  |  | x | x |  |  | x |
| Overnight (after) | x | |  | |  | x | x |  |  | x |  |  |

^a^FTX: field training exercise.

^b^HRV: heart rate variability.
