# Supplementary figures and images for: Intensive, Real-Time Data Collection of Psychological and Physiological Stress During a 96-Hour Field Training Exercise at a Senior Military College: Feasibility and Acceptability Cohort Study
Source: JMIR Form Res. 2024 Oct 18;8:e60925. doi: 10.2196/60925 (PMC11530722; doi:10.2196/60925)

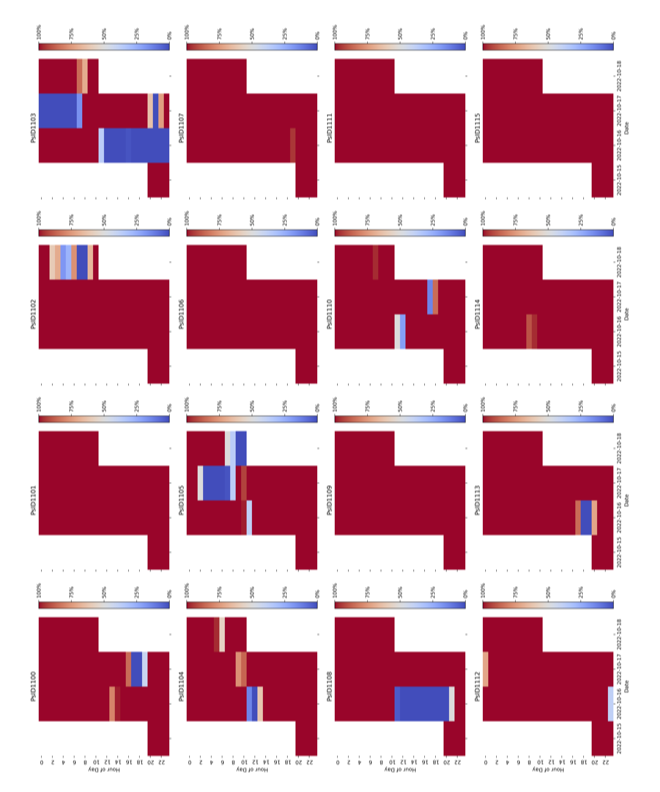

Supplement: Multimedia Appendix 2 [file formative_v8i1e60925_app2.png]
