# Supplementary material for: Intensive, Real-Time Data Collection of Psychological and Physiological Stress During a 96-Hour Field Training Exercise at a Senior Military College: Feasibility and Acceptability Cohort Study
Source: JMIR Form Res. 2024 Oct 18;8:e60925. doi: 10.2196/60925 (PMC11530722; doi:10.2196/60925)
Supplement: Multimedia Appendix 3 [file formative_v8i1e60925_app3.docx]

**Table S1.**

| **Acceptability prompt (n=)** | **Mean (SD)** |  | **Mean (SD)** |  | **Mean (SD)** |
| --- | --- | --- | --- | --- | --- |
| **OURA (n=9)** |  | **EKG (n=4)** |  | **Sweat sensor (n=8)** |  |
| I liked wearing OURA | 4.4 (0.97) | I liked wearing the EKG | 3.5 (0.58) | I liked wearing the sweat sensor | 4.14 (1.07) |
| OURA was physically uncomfortable | 2.67 (1.86) | The EKG was physically uncomfortable | 3.5 (1.29) | The sweat sensor was physically uncomfortable | 3.4 (1.52) |
| Wearing OURA was embarrassing | 1.0 (0.0) | Wearing EKG was embarrassing | 1.5 (0.71) | Wearing sweat sensor was embarrassing | 1.25 (0.5) |
| OURA interfered with my regular activities such as school, work, FTX participation, etc | 1.25 (0.5) | The EKG interfered with my regular activities such as school, work, FTX participation, etc | 2.5 (0.71) | The sweat sensor interfered with my regular activities such as school, work, FTX participation, etc | 1.75 (0.96) |
| OURA fell off easily during my daily activities/sleep | 1.5 (0.71) | EKG fell off easily during my daily activities/sleep | 3.33 (1.53) | The sweat sensor fell off easily during my daily activities/sleep | 1.33 (0.58) |
| OURA interfered with my exercise | 1.75 (0.96) | The EKG interfered with my exercise | 3.33 (0.58) | The sweat sensor interfered with my exercise | 2.25 (1.5) |
| OURA interfered with my sleep | 1.0 (±0.0) | The EKG interfered with my sleep | 4.0 (0.0) | The sweat sensor interfered with my sleep | 1.75 (0.96) |
| OURA interfered with my ability to concentrate | 1.0 (0.0) | The EKG interfered with my ability to concentrate | 2.0 (1.0) | The sweat sensor interfered with my ability to concentrate | 1.67 (0.58) |
| OURA interfered with my choice of jewelry/accessories | 1.0 (0.0) | The EKG interfered with my choice of jewelry/accessories | 3.0 (0.0) | The sweat sensor interfered with my choice of jewelry/accessories | 1.33 (0.58) |
| I often forgot I was even wearing OURA | 4.38 (0.74) | I often forgot I was even wearing the EKG | 2.5 (0.71) | I often forgot I was even wearing the  sweat sensor | 3.14 (1.21) |
| I did not have to change my daily routine (or give anything up) in order to comply with wearing OURA | 4.89 (0.74) | I did not have to change my daily routine (or give anything up) in order to comply with wearing the EKG | 3.5 (1.29) | I did not have to change my daily routine (or give anything up) in order to comply with wearing the sweat sensor | 3.33 (1.51) |
| I would be willing to wear OURA for longer than I was asked | 4.78 (0.67) | I would be willing to wear the EKG for longer than I was asked | 3.5 (2.12) | I would be willing to wear the sweat sensor for longer than I was asked | 4.29 (1.25) |
| I would be willing to wear OURA again in the future | 4.9 (0.32) | I would be willing to wear the EKG again in the future | 4.0 (1.41) | I would be willing to wear the sweat sensor again in the future | 4.25 (1.16) |
| I found it difficult to remember to wear or charge OURA | 2.0 (1.73) |  |  |  |  |
| I had difficulty linking the app and the OURA ring properly | 1.83 (0.41) |  |  |  |  |
